# Maintenance of Fluorescence During Paraffin Embedding of Fluorescent Protein-Labeled Specimens

**DOI:** 10.3389/fnins.2019.00752

**Published:** 2019-07-23

**Authors:** Ouyang Zhanmu, Peilin Zhao, Yang Yang, Xiaoquan Yang, Hui Gong, Xiangning Li

**Affiliations:** ^1^Britton Chance Center for Biomedical Photonics, Wuhan National Laboratory for Optoelectronics-Huazhong University of Science and Technology, Wuhan, China; ^2^MoE Key Laboratory for Biomedical Photonics, School of Engineering Sciences, Huazhong University of Science and Technology, Wuhan, China; ^3^HUST-Suzhou Institute for Brainsmatics, Suzhou, China

**Keywords:** paraffin embedding, green fluorescent protein, dehydration, preservation of fluorescence, whole brain

## Abstract

Paraffin embedding is widely used in microscopic imaging for preparing biological specimens. However, owing to significant fluorescence quenching during the embedding process, it is not compatible with fluorescent-labeling techniques, such as transgenic and viral labeling using green fluorescent protein (GFP). Here, we investigate the quenching mechanism and optimize the embedding process to improve the preservation of fluorescence intensity. The results show that dehydration is the main reason for fluorescence quenching during paraffin embedding, caused by the full denaturation of GFP molecules in ethyl alcohol. To evaluate fluorescent and morphological preservation, we modified the embedding process using tertiary butanol (TBA) instead of ethyl alcohol. Fluorescence intensity following TBA dehydration increased 12.08-fold of that observed in the traditional method. We obtained uniform fluorescence maintenance throughout the whole mouse brain, while the continuous apical dendrites, spines, and axon terminals were shown evenly within the cortex, hippocampus, and the amygdala. Moreover, we embedded a whole rat brain labeled with AAV in the prelimbic cortex (Prl). With the axon terminals in different areas, such as the caudate putamen, thalamus, and pyramidal tract, the results showed a continuous tract of Prl neurons throughout the whole brain. This method was also suitable for tdTomota labeled samples. These findings indicate that this modified embedding method could be compatible with GFP and provides a potential turning point for applications in the fluorescent labeling of samples.

## Introduction

Paraffin embedding is a well-developed method that is widely used as a basic tool for histomorphology. Compared to other methods, embedding shows good morphological preservation, and sectioning properties (Onodera et al., 1992), but unfortunately quenches fluorescent proteins. This results in a poor fluorescent signal that makes detection near impossible. Green fluorescent protein (GFP) and its variants, including the widely used enhanced green fluorescent proteins (EGFP) and enhanced yellow fluorescent proteins (EYFP), are used in many applications, such as neuroscience, drug screening, and cancer research. For example, transgene and virus tracing with GFP-based reporter proteins provides a convenient way to explore neuronal morphology (Li et al., 2017, 2018), disease progression (Kammertoens et al., 2017), and the structure and function of neural circuits (Kim et al., 2015; Yao et al., 2018; Zhou et al., 2018). In order to combine paraffin embedding with fluorescent labeling techniques, antibodies are employed to detect GFP proteins as a compromise (Vankelecom, 2009; Porter et al., 2017; Ikeda, 2018). However, this compromising staining method is tedious and may result in either false negatives or false positives (True, 2008). Thus, the development of a paraffin embedding method compatible with fluorescent protein labeling is urgently needed.

Most researchers believe that the chemical fixing reagents and high temperatures used in the process of paraffin embedding results in the quenching of GFP and its variants (Jiang et al., 2005; Swenson et al., 2007; Smith et al., 2015). It is generally agreed that the most effective solution to detect paraffin embedded GFP is to stain them by immunofluorescence or immunohistochemistry (IHC). However, the specific reasons for fluorescence quenching during paraffin embedding remains inferred. Since GFP has been used to label biological molecules and structures (Kain et al., 1995), the requirement to combine this with traditional histochemical methods has emerged. Researchers have attempted to improve the preservation of fluorescence in paraffin embedded samples by empirically optimizing the embedding protocol (Vankelecom, 2009; Porter et al., 2017). The most successful improvement used a modified version using an ethanol fixation at 4°C, which allows GFP visualization (Nakagawa et al., 2015). However, not all samples are effectively fixed when using ethanol and, while GFP could be detected, no details for fine structure were shown. Therefore, fluorescence quenching still exists, making it difficult to consider the application of GFP in paraffin embedding.

To address this problem, essential questions remain to be answered: what happens to fluorescent protein molecules during paraffin embedding that causes significant fluorescence quenching? Can fluorescence quenching be effectively avoided? In this study, we answer these questions by tracing the behaviors of GFP during the paraffin embedding process and develop a modified paraffin embedding method for fluorescence preservation. Our results show that GFP molecules are fully damaged in ethyl alcohol (EtOH), resulting in fluorescence quenching. A modified version using tertiary butanol (TBA) dehydration increased fluorescent intensity 12.08-fold compared to the traditional embedding method. GFP-labeled murine brain sections embedded in paraffin showed that the amount of signal and morphological detail were both perfectly preserved; we not only acquired sophisticated morphological neuron structures, but also the distribution of local and long-range projections. This modified method is considered to overcome the limits of traditional paraffin embedding in relation to fluorescent labeling and provides for additional study applications.

## Materials and Methods

### Animals

Eight-week-old Thy1-GFP-M, Thy1-YFP-H, Chat-cre::Ai14 mice and Wistar rats were used in this study. Thy1-GFP-M, Thy1-YFP-H line, Chat-cre and Ai14 mice were acquired from the Jackson Laboratory. Wistar rats were acquired from the Hubei Provincial Center for Disease Control and Prevention. Animal care and use was performed in accordance with the guidelines of the Administration Committee of Affairs Concerning Experimental Animals in Hubei Province of China. The protocol was approved by the Committee on the Ethics of Animal Experiments of the Huazhong University of Science and Technology. All surgery was performed under sodium pentobarbital anesthesia, and every effort was made to minimize animal suffering.

### Viral Labeling

AAV2/9-EGFP (BrainVTA) was used as the anterograde tracer. The stereotaxic coordinates for the target areas were based on the Rat Brain in Stereotaxic Coordinates Atlas. Using a pressure injector (Nanoject II; Drummond Scientific Co., Broomall, PA, United States), 150 nl of AAV-EGFP was injected into the prelimbic cortex (Prl) of an 8-week-old SD rat (5.10 mm A-P, 1.00 mm medial-lateral, and 3.60 mm dorsal-ventral).

### Preparation of Brain Sections

Mice were anesthetized with a 1% solution of sodium pentobarbital and perfused with 10 mM phosphate buffered saline (PBS, Sigma) followed by 4% PFA (Sigma-Aldrich) in 10 mM PBS. The entire brain was removed and post-fixed in 4% PFA at 4°C for 12 h. After fixation, the mouse brain was rinsed overnight at 4°C in 10 mM PBS. The 20 μm-thick brain slices (fresh) were acquired using a vibration microtome (Leica, VT1000 S). The 10 μm or 20 μm-thick brain slices (paraffin embedded) were collected by a fully motorized rotary microtome (Leica, RM2250).

### Traditional Paraffin Embedding

For mouse brains, the brain was first removed from the skull and transferred to neutral buffered PFA (4% w/v in 10 mM PBS, 50 ml per brain) for 24 h post fixation. Following fixation, the mouse brain was transferred to fresh 10 mM PBS for a 12–24 h rinse (50 mL per mouse brain). A graded series of ethanol solutions were then used to dehydrate the tissue (five solutions were used: 50, 75, 95, 100, and 100%, v/v, each incubated for 2 h at 30°C). After dehydration, the samples were ready for infiltration: they were successively soaked in a graded series of infiltration solutions (50% in 100% ethanol and 100% in 100% xylene, 1 h for the first solution, 0.5 h for the second, and 0.5–1 h for the final solution at 30°C). The brain tissue should be rendered totally transparent by the xylene. Specimens were then immersed in paraffin at 60°C for 12 h (paraffin was changed every 4 h). For brain hemispheres, all treating times could be halved.

### TBA-Dehydrating Paraffin Embedding

For mouse brains, the brain was first removed from the skull and transferred to neutral buffered PFA (4% w/v in 10 mM PBS, 50 ml per brain) for 24 h post fixation. Following fixation, the mouse brain was transferred to fresh 10 mM PBS for a 12–24 h rinse (50 ml per mouse brain). A graded series of TBA solutions were then used to dehydrate the tissue (five solutions were used: 50, 75, 95, 100, and 100%, each incubated for 12 h at 30°C). After dehydration, the samples were immersed in paraffin at 60°C for 12 h (paraffin was changed every 4 h). For brain hemispheres, all treating times could be halved.

For rat brains, the brain was first removed from the skull and transferred to neutral buffered PFA (4% w/v in 10 mM PBS, 100 ml per brain) for 24 h post fixation. Following fixation, the rat brain was transferred to fresh 10 mM PBS for a 24–36 h rinse (100 ml per brain). A graded series of TBA solutions were then used to dehydrate the tissue (five solutions were used: 50, 75, 95, 100, and 100%, each incubated for 24 h at 30°C). After dehydration, the samples were immersed in paraffin at 60°C for 36 h (paraffin was changed every 4 h). For brain hemispheres, all treating times could be halved.

### Absorption Spectrometry

Recombinant EGFP was a histidine-tagged fusion protein of GFP expressed in *Escherichia coli* and purified by nickel-chelate affinity chromatography (Alkaabi et al., 2005). All absorption spectra were measured on a LAMBDA 950 UV/Vis/NIR Spectrophotometer (PerkinElmer), with a 1 nm spectral resolution. The recombinant EGFP solution was dialyzed in distilled water to remove all salts. Absorption spectra of recombinant EGFP (ddH_2_O, pH = 7.0) was measured against the controls. Recombinant EGFP was then diluted to 0.25 mg/ml with dehydrating agents. All absorption spectra were normalized at the 429 nm isosbestic point measured for standard solution conditions for further analysis.

### Fluorescent Imaging

Evaluation of the lye-activated paraffin embedded fluorescent protein-labeled tissue used 10 μm thick mouse brain paraffin sections that were collected and dried at 56°C. A commercial confocal microscope (Zeiss, LSM710) was configured to step scan the tissue sections at 1 μm per slice to acquire a 10 μm z-stack at 25°C.

### Preservation Analysis

Neuron fluorescent intensity in images was analyzed using ImageJ software. First, an oval area was selected in the soma of a neuron with the limited brain area. Next, using the histogram tool, the mean fluorescent intensity of the oval area was measured to reflect the fluorescent intensity of the neuron. The fluorescent intensity of 30–50 neurons was counted in a single sample, and the average value calculated to reduce error. Assuming the average fluorescent intensity of the control group was “A,” and the fluorescent intensity of the experimental group was “B,” the fluorescent preservation of one sample, named “C,” was calculated as follows: C = B/A × 100%. For each group, at least five samples were analyzed and the final value for fluorescent preservation calculated as C_o_ = (C_1_+C_2_+...+C_n_)/n (n represents the total number of samples).

### Statistics

All statistical graphs were generated using GraphPad Prism 7.01. The two-tailed student’s *t*-test was also performed using GraphPad Prism 7.01. The confidence level was set to 0.05 (*P*-value), and all results are presented as the mean ± SD.

## Results

### Mechanism of EGFP Fluorescence Quenching During Paraffin Embedding

**FIGURE 1:**
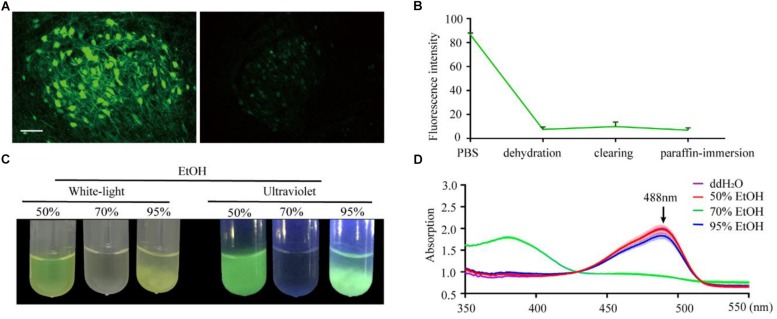
Changes in EGFP fluorescence during paraffin embedding. **(A)** Images of fluorescent samples before and after embedding. All images were maximum intensity projections of 20 μm thick z-stacks and acquired on the same microscope (Zeiss, LSM710) with the same configuration at 25°C (room temperature). **(B)** The statistical graph of fluorescent intensity during paraffin embedding. Values are mean ± *SD*; *n* = 3 for each group plotted. **(C)** Images of EGFP fluorescence in 50, 70, and 95% EtOH (v/v) under white-light and ultraviolet, respectively. **(D)** The absorption spectrum of EGFP in 50, 70, and 95% EtOH (v/v); the concentration of EGFP in the dehydrating agents was 0.25 mg/ml, *n* = 3. Scale bars, 100 μm.

To confirm the effect of the paraffin embedding process on endogenous fluorescent signals, we embedded mouse brains expressing GFP in neurons (Thy1-GFP-M mice). For each brain, the right hemisphere was set as the control group while the left hemisphere was embedded. As shown in Figure 1A, GFP fluorescence could still be detected following embedding but was significantly quenched compared to the control group. To explore the key factors causing fluorescence quenching, we used an assay based on purified recombinant EGFP (histidine-tagged, expressed in *E. coli*) embedded in agarose, which allowed for quantification of fluorescence to be monitored throughout the embedding process. The majority of loss in fluorescence was found to occur during the dehydration steps (91.4% ± 2.4% decreased after dehydration, 2.8% ± 2.1% recover after clearing, 3.5% ± 2.3% loss after paraffin-immersion, Figure 1B).

To further investigate what caused quenching of fluorescence protein, EGFP were dissolved into an EtOH gradient and kept at room temperature for 1 h; as per the dehydrating procedure in the normal paraffin embedding process for the mouse brain. The results showed that 70% EtOH caused significant fluorescence quenching (Figure 1C). This raised the question of whether GFP was directly damaged or changed to a non-fluorescent state during the dehydrating process. To address this, we measured the absorption spectrum of recombinant EGFP. As EGFP proteins were insoluble in the 95% dehydrating agents (Figure 1C), the supernatant liquid was discarded after centrifugation and the precipitate dissolved by the same volume of ddH_2_O before measuring the absorption spectrum. The absorption spectrum of EGFP, with peaks at 380 nm, was detected in 70% EtOH (Figure 1D), indicating denatured or digested EGFP (Cody et al., 1993). No shoulders of absorption were observed around 380 nm and 448 nm in 50% EtOH and 95% EtOH, suggesting full EGFP denaturation could be neglected in these agents (Figure 1D). Thus, fluorescence quenching during embedding appeared to mainly be the result of damage caused by 70% EtOH during dehydration.

### Fluorescence Retention During TBA Dehydration

**FIGURE 2:**
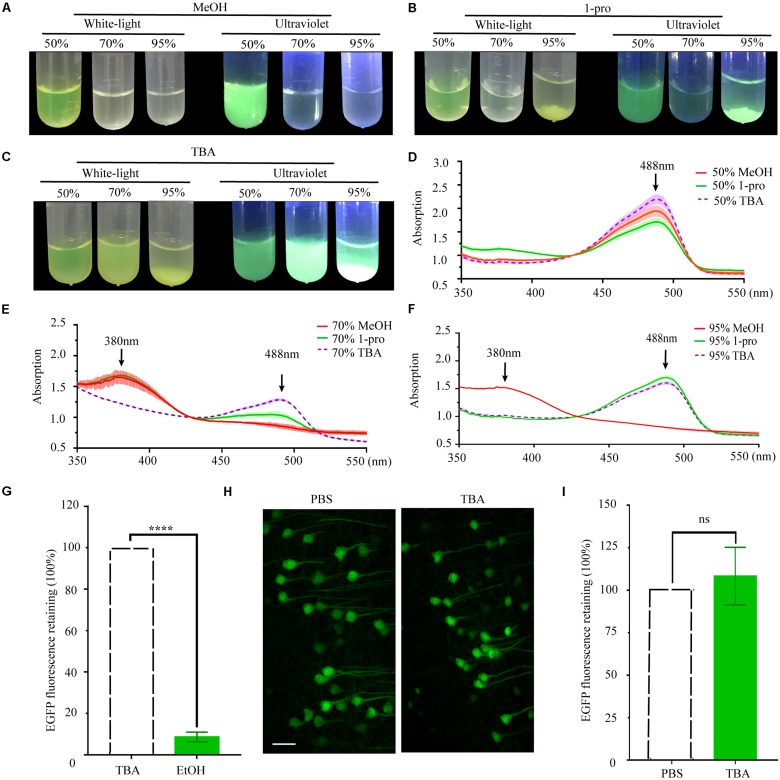
Changes and retention of EGFP fluorescence during dehydration. **(A)** Images of EGFP fluorescence in 50, 70, and 95% MeOH (V/V) under white-light and ultraviolet, respectively. **(B)** Images of EGFP fluorescence in 50, 70, and 95% 1-pro (V/V) under white-light and ultraviolet, respectively. **(C)** Images of EGFP fluorescence in 50, 70, and 95% TBA (V/V) under white-light and ultraviolet, respectively. **(D)** The absorption spectrum of EGFP in 50% MeOH, 50% 1-pro, and 50% TBA (V/V); **(E)** The absorption spectrum of EGFP in 70% MeOH, 70% 1-pro, and 70% TBA (V/V); **(F)** The absorption spectrum of EGFP in 95% MeOH, 95% 1-pro, and 95% TBA (V/V); Concentration of EGFP in dehydrating agents was 0.25 mg/ml, *n* = 3. **(G)** Fluorescence intensity statistics in fluorescent hemispheres. The left hemisphere was dehydrated with EtOH and embedded with paraffin. The right hemisphere was dehydrated with TBA and then embedded with paraffin as a control. mean ± *SD*; *n* = 5 brains for each group plotted. Statistical significance (^*⁣*⁣**^P < 0.0001 was assessed by unpaired *t*-test). **(H)** Images of fresh and paraffin-embedded fluorescent hemispheres. The left hemisphere was embedded and sectioned into 20 μm-thick paraffin slices. The right hemisphere was sectioned into 20 μm-thick fresh slices. **(I)** Fluorescence intensity statistics in **(F)**. Scale bars, 50 μm; mean ± SD; *n* = 6 brains for each group plotted. Statistical significance (*P* > 0.5) was assessed by unpaired *t*-test.

As per previous reports, the length of the carbon chain and branch number of the hydrocarbon portion of alcohols affect their effectiveness as protein denaturants (Sirotkin et al., 1997). To compare the effect of different hydrocarbon portions, we used methyl alcohol (MeOH), propyl alcohol (1-pro), TBA as the dehydrating agents. EGFP proteins were dissolved into gradient dehydrating agents and kept at 30°C for 1 h. Fluorescence quenched significantly in 70% MeOH, 95% MeOH, and 70% 1-pro (Figures 2A,B), while fluorescence did not significantly change in different gradients of TBA solution (Figure 2C). Then the absorption spectrum of recombinant EGFP was measured. EGFP proteins dissolved in 95% MeOH but were insoluble in 95% 1-pro and 95% TBA. To certify what happened to the protein during the precipitation process, we collected the deposits of EGFP in 95% 1-pro and 95% TBA and dissolved them in ddH_2_O again. The absorption spectrum of these precipitated EGFP had a peak at 380 nm in 70% MeOH and 95% MeOH (Figures 2D–F), while only one peak was detected at 488 nm in graded TBA solutions (Figures 2D–F). There were two peaks at 380 and 488 nm in 70% 1-pro, which indicated that a part of proteins was denatured or digested (Figures 2D–F). Then, we compared the EGFP intensity of 100 μm-thick brain sections from Thy1-EGFP-M mice before and after dehydrating with MeOH, EtOH, 1-pro, and TBA, respectively. Fluorescent intensity significantly decreased in the MeOH, EtOH, and 1-pro experiment group, and increased in the TBA-group (Supplementary Figure S1). Enhancement of fluorescent intensity was mainly due to cell shrinkage caused by dehydration. These results demonstrated that more fluorescence was retained after dehydrating with TBA, which was consistent with that in the GFP protein group (Figures 2A–F).

As the complete protocol for paraffin embedding was more complex than the dehydration protocol alone, analysis of the dehydration steps could not be representative of final fluorescence preservation. Preservation of fluorescence still needed to be assessed in paraffin embedded tissues. The left hemispheres of PFA fixed thy1-EGFP-M mouse brains were dehydrated in a graded series of EtOH, while the corresponding right hemispheres were dehydrated in a graded series of TBA as the control. All hemispheres were subsequently embedded and fluorescence signals quantitatively investigated in the left and right hemispheres and compared. Our results demonstrated that TBA significantly increased fluorescence intensity compared with EtOH (12.08 ± 3.27-fold increase, *n* = 6 slices for one mouse, five mice per group, ^*⁣*⁣**^P < 0.0001, Figure 2G and Supplementary Figure S2). To further assess the compatibility of GFP and its mutant proteins with TBA dehydration of the whole brain during the paraffin embedding process, we quantitatively compared EYFP fluorescent signals before and after embedding. The left hemispheres were embedded following dehydration of the whole brain, while the corresponding right hemispheres were sectioned into fresh slices as a control. A slight increase of GFP fluorescence was observed after embedding (108.3 ± 16.9% retained, *n* = 8 slices for one mouse, six mice per group, *P* > 0.5, Figures 2H,I).

### Structure Preservation and Fluorescence Retention

**FIGURE 3:**
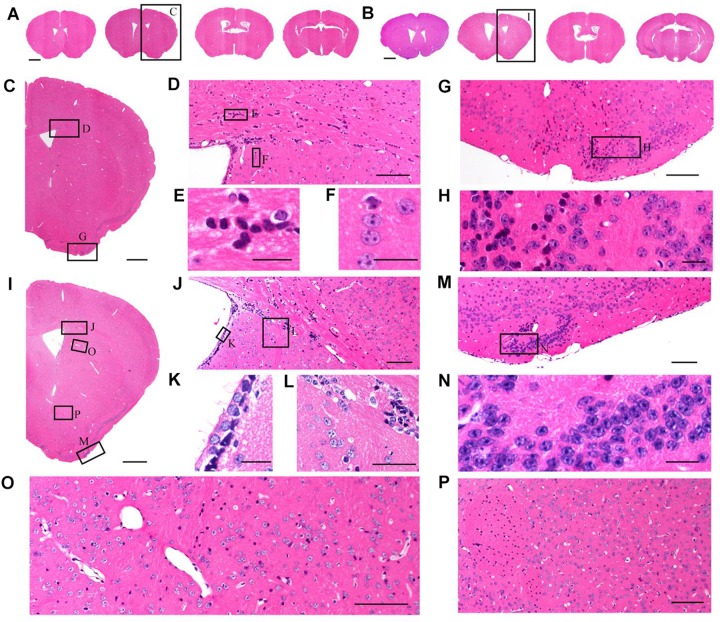
Comparison of brain morphological preservation. **(A)** Images of 5 μm-thick mouse brain paraffin section stained by HE. EtOH was used as the dehydrating agent. **(B)** Images of 5 μm-thick mouse brain paraffin section stained by HE. TBA was used as the dehydrating agent. **(C)** An enlarged view of the area indicated by the rectangle in **(A)**. **(D)** An enlarged view of the area indicated by the rectangle in **(C)**. **(E,F)** An enlarged view of the area indicated by the rectangle in **(D)**. **(G)** An enlarged view of the area indicated by the rectangle in **(C)**. **(H)** An enlarged view of the area indicated by the rectangle in **(G)**. **(I)** An enlarged view of the area indicated by the rectangle in **(B)**. **(J)** An enlarged view of the area indicated by the rectangle in **(I)**. **(K,L)** An enlarged view of the area indicated by the rectangle in **(J)**. **(M)** An enlarged view of the area indicated by the rectangle in **(I)**. **(N)** An enlarged view of the area indicated by the rectangle in **(M)**. **(O,P)** An enlarged view of the area indicated by the rectangle in **(I)**. Scale bars, **(A,B)** 1 mm; **(C)** 500 μm; **(D)** 100 μm; **(E,F)** 20 μm; **(G)** 100 μm; **(H)** 20 μm; **(I)** 500 μm; **(J)** 100 μm; **(K,L)** 20 μm; **(M)** 100 μm; **(N)** 20 μm; and **(O,P)** 100 μm.

Although TBA-dehydration improved fluorescence preservation compared to EtOH-dehydration, it remained unknown whether this modification influenced the brain structure and cellular morphology. Here, we used HE staining to compare the cytoarchitectonic structure of C57 mice embedded in paraffin. Brains were dehydrated by EtOH and TBA, respectively, and embedded in paraffin. Then, 5 μm-thick paraffin sections were collected and stained by HE. The results showed that the paraffin embedded sections were intact and stained uniformly (Figures 3A,B). From the HE staining figures, we can distinguish the cellular nuclei morphology clearly (Figures 3C–P). As shown in Figure 3, we found that the brain and cell structure was preserved without significant differences compared with that of traditional paraffin embedding (dehydrating by EtOH), indicating that our method is feasible.

**FIGURE 4:**
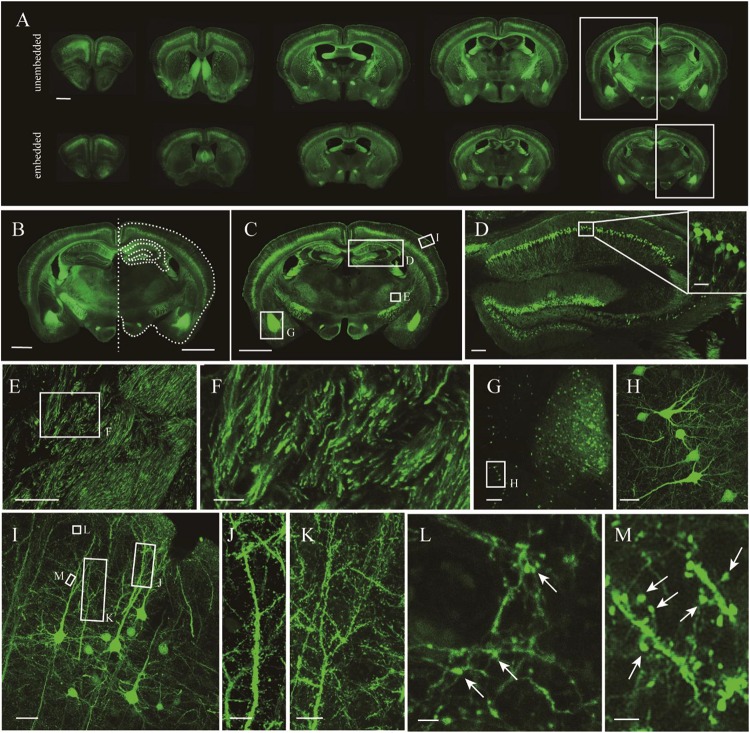
Fluorescence retention and preservation of neuronal morphology and fine structure. **(A)** Images of fresh and paraffin embedded sections. **(B)** Matching image of the area indicated by the rectangle in **(A)**. Right side of the fresh section appears as the white outline. **(C)** Image of a coronal plane. **(D,E)** An enlarged view of the area indicated by the rectangle in **(C)**. **(F)** An enlarged view of the area indicated by the rectangle in **(E)**. **(G)** An enlarged view of the area indicated by the rectangle in **(C)**. **(H)** An enlarged view of the area indicated by the rectangle in **(G)**. **(I)** An enlarged view of the area indicated by the rectangle in **(C)**. **(J–M)** An enlarged view of the area indicated by the rectangle in **(I)**, demonstrating the visualization of axonal boutons, which are indicated using arrowheads. Scale bars, **(A–C)** 1 mm; **(D)** 100 μm, white box, 100 μm; **(E)** 100 μm; **(F)** 20 μm; **(G)** 100 μm; **(H)** 20 μm; **(I)** 100 μm; **(J,K)** 10 μm; and **(L,M)** 2 μm.

Given that the uniformly high preservation ratios of EYFP and EGFP mice enabled reliable detection of labeled structures in paraffin embedded tissue, we first compared whole adult mouse brains. A comparison of similar parts indicated uniform shrinkage in embedded specimens, but importantly no significant deformation was detected (Figure 4A). The paraffin section could be perfectly matched with fresh sections (Figure 4B). As such, the fine structures in paraffin sections were measured (Figure 4C). The results showed that apical dendrites, spines, and axon boutons could be distinguished in the respective brain regions, including the hippocampus, cortex, and the amygdala (Figures 4D–M). These meticulous comparisons of the fine structures showed that signal intensity and complicated morphological detail were both perfectly preserved following TBA dehydration. Moreover, TBA did not influence immunohistochemical (IHC) and HE staining (Supplementary Figure S3). Since more GFP proteins were retained, we could simultaneously acquire the original and IHC-stained fluorescent signals (Supplementary Figure S3).

**FIGURE 5:**
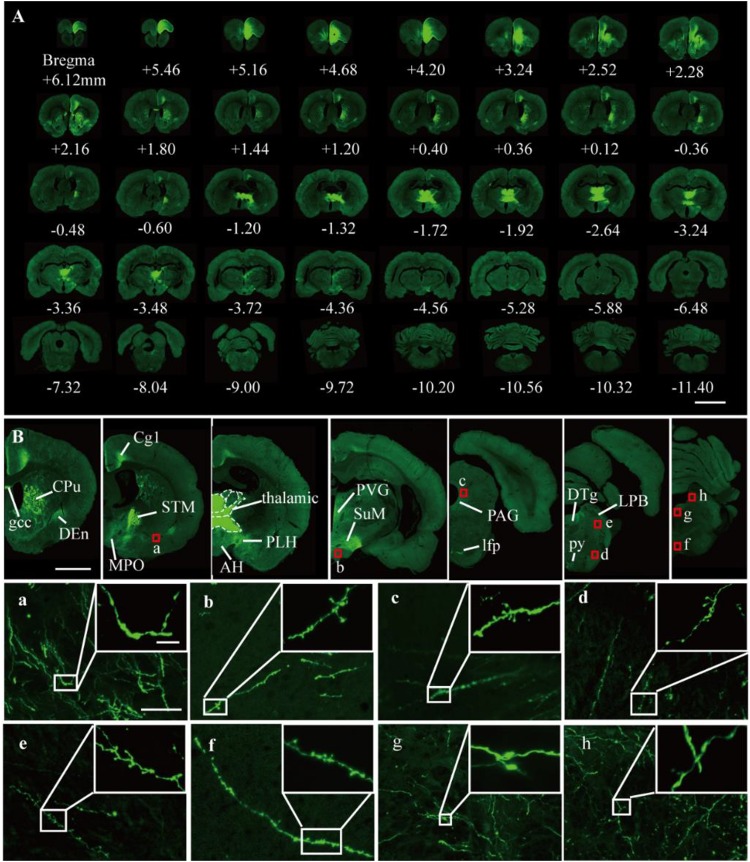
Fluorescent images of viral tracing in adult rats. **(A)** Slices were placed on slides and manually imaged with a Zeiss LSM710 confocal microscope. **(B)** Neural projections of different coronal planes were shown. **(a–h)** An enlarged view of the area indicated by the red rectangle in **(B)**. Scale bars, **(A)** 5 mm; **(B)** 2 mm; **(a–h)** 50 μm; and **(a–h)** white box, 5 μm. gcc, Genu of the corpus callosum; Cg, cingulate cortex; CPu, caudate putamen; Den, dorsal endopiriform nucleus; MPO, medial preoptic nucleus; PLH, peduncular part of lateral hypothalamus; AH, anterior hypothalamic area; STM, bed nucleus of the stria terminalis; PVG, periventricular gray; SuM, supramammillary nucleus; PAG, periaqueductal gray; lfp, longitudinal fasciculus of the pons; LPB, lateral parabrachial nucleus; DTg, dorsal tegmental nucleus; py, pyramidal tract.

**FIGURE 6:**
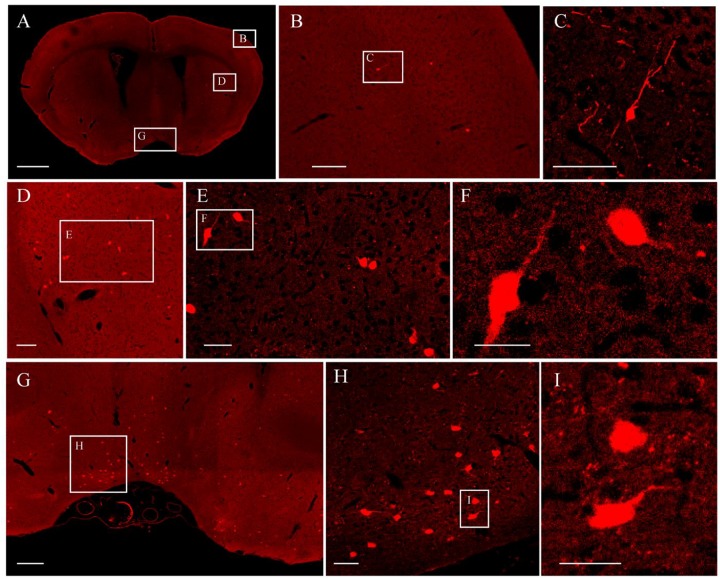
The fluorescent images of tdTomato-labeled brain. **(A)** Image of a coronal plane. **(B)** An enlarged view of the area indicated by the rectangle in **(A)**. **(C)** An enlarged view of the area indicated by the rectangle in **(B)**. **(D)** An enlarged view of the area indicated by the rectangle in **(A)**. **(E)** An enlarged view of the area indicated by the rectangle in **(D)**. **(F)** An enlarged view of the area indicated by the rectangle in **(E)**. **(G)** An enlarged view of the area indicated by the rectangle in **(A)**. **(H)** An enlarged view of the area indicated by the rectangle in **(G)**. **(I)** An enlarged view of the area indicated by the rectangle in **(H)**. Scale bars, **(A)** 1 mm; **(B)** 100 μm; **(C)** 50 μm; **(D)** 100 μm; **(E)** 50 μm; **(F)** 25 μm; **(G)** 200 μm; **(H)** 50 μm; and **(I)** 25 μm.

It was further demonstrated that the TBA-dehydrating paraffin embedding method could also be used in large tissues labeled with fluorescent protein as well as whole rat brains. Here, anterograde projection mapping of the prelimbic cortex (PrL) in an adult rat was performed. To label neurons whose somas were located in the PrL, AAV 2/9-EGFP (de Solis et al., 2017) was injected. EGFP-labeled fibers were successfully distinguished in the whole brain, with fluorescent signals of neuronal projections intensely observed in the prefrontal cortex, thalamic, hypothalamus, and regions of the midbrain (Figures 4A, 5A). The distance and connection strength of these projections varied between different areas (Figures 4B, 5A). The pyramidal tract (py) was the farthest region where projections reached, while the connection strength with the PrL was the weakest. Importantly, neuronal fibers could still be detected and distinguished (Figures 4B, 5B). Projections from the PrL could be traced to the py due to continuous paraffin sections and fine fluorescent signals. Projections from the PrL mainly projected successively through the genu of the corpus callosum, cingulate cortex, peduncular part of the lateral hypothalamus, a number of thalamic nuclei, py, and so on (Figures 4B, 5B). These results indicate that the PrL has direct connections with multiple brain regions and plays an important role in normal physiological activities. In addition, due to its high fluorescent retention, fine images of small and weakly labeled structures, such as the axon terminals, could be provided (Figure 5B). To confirm whether this method was suitable for other fluorescent proteins, we embedded the brains of Chat-IRES-Cre::Ai14 mice, which were crossed with the Chat-ires-cre mice with Ai reporter line (Li et al., 2018). In these brains, the cholinergic neurons were labeled with tdTomato. After being embedded in paraffin, 10 μm-thick sections were collected and imaged with a confocal microscope (Figure 6A). The soma of cholinergic and the dendrites around the soma were visible in the cortex (Figures 6B,C), caudate putamen (Figures 6D–F) and basal forebrain (Figures 6G–I). These results demonstrates that our optimized embedding method could be used in tdTomato.

## Discussion

In this study, we explored the mechanism of GFP fluorescence quenching during paraffin embedding and developed an optimized protocol based on a mechanism we have called TBA-dehydrating paraffin embedding. Evaluation of the traditional embedding procedure found that most GFP molecules are directly damaged by EtOH. TBA is a more GFP-friendly organic agent that does not disrupt GFP structure. This improved method allows for paraffin embedding to be compatible with other powerful analytical techniques based on GFP, while still retaining the advantages of embedding, such as uniform size reduction, semi-thin sectioning, morphological preservation, HE staining, and IHC staining.

Perhaps the most significant discovery described herein is confirmation of the mechanism of fluorescence quenching in paraffin embedded tissues. In previous studies, only IHC-enabled methods could offer a compromise to successfully detect GFP fluorescence in paraffin embedded samples (Walter et al., 2000; Boeloni et al., 2010). Only a few studies have focused on evaluating the fixation and temperature used in paraffin immersion (Vankelecom, 2009; Nakagawa et al., 2015) for direct imaging of fluorescence. However, these methods did not provide fine structures during EGFP quenching. Without the guidance of an identified mechanism describing the behaviors of fluorescent protein molecules during paraffin embedding, only limited success can be achieved following previous methods for macroscopic tissue embedding. Dehydration was found to be the main reason for fluorescence quenching (Figures 1A,B), with two possible explanations provided. One is that EtOH induces most GFP molecules to transform to a non-fluorescent state. The other is that GFP is directly damaged by EtOH. Faced with this possibility, GFP should be protected from destruction. Our absorption spectrum study shows that the second explanation for fluorescence quenching is correct. Full EGFP denaturation occurred in 70% EtOH, with most EGFP chromophores being damaged (Figures 1C,D). It is well known that GFP only emits fluorescence when it has the correct tertiary structure of its native form. When GFP is fully denatured, the chromophore is attacked, and its fluorescence quenched. This irreversible chromophore denaturation results in the permanent loss of visible fluorescence (Niwa et al., 1996; Ormo et al., 1996; Arpino et al., 2012). In addition, alcohols damage the native protein structure in aqueous solution by changing the solvent’s dielectric constant (Cinelli et al., 1997). This may explain why chromophore damage only appeared in 70% EtOH.

Much work has been done to study protein denaturation by alcohols, but limitations in this area remain. Proteins used in previous studies were usually non-luminescent, such as HSA (Sirotkin et al., 1997), globule proteins (Kundu et al., 2017), and chymotrypsin inhibitor (Mohanta and Jana, 2016). No experiments about the relationship between GFP proteins and alcohols have been performed. As per previous reports, the length of the carbon chain and branching of the hydrocarbon portion of alcohols tends to reduce their effectiveness as protein denaturants (Sirotkin et al., 1997). Because alcohols with more than five carbon atoms have poor compatibility with water, we used MeOH, EtOH, 1-pro, and TBA as the dehydration solution. Compared with MeOH, EtOH, and 1-pro, the longer carbon chain and two branches make TBA friendly to GFP proteins. Our results show that fluorescence was fully quenched in 70% MeOH, 95% MeOH, and 70% EtOH, while partly quenched in 70% 1-pro (Figures 2A–F). Additionally, the absorption spectrum results also showed chromophores were only intact in the graded TBA solution (Figures 2D–F).

This improved method allows for the visualization of natural GFP fluorescence and offers several advantages over traditional paraffin embedding methods. First, TBA-dehydration increases fluorescent intensity and enables direct imaging of paraffin embedded GFP proteins without any noticeable loss of labeled structures, such as high-resolution images of the fine structures of layer V pyramidal neurons in whole adult mouse brains (Figures 3–5). Although not as good as GFP, our method can also be used to embed tdTomato proteins. Second, our method enables high fluorescent retention for complete fluorescence analysis, while simultaneously maintaining tissue histology (Figures 2–4). Due to generally poor fluorescent preservation, traditional paraffin embedding is often restricted to morphological and histological studies and therefore requires additional experimental animals for fluorescence analysis. Third, our method overcomes the restriction of sample volumes. In this study, we used our method to demonstrate long-range neuronal projection and axonal boutons in the whole adult rat brain (Figure 4). The total tissue volume available was about a factor of approximately 2–3 times larger than for conventional methods. Long treatment times did not influence fluorescence intensity. The method can be applied to entire tissues. Moreover, tissues embedded by this method allow detection of the original fluorescence after IHC staining because of the increased retention of GFP fluorescence (Supplementary Figure S3). Therefore, it could serve as a useful method to simultaneously acquire natural GFP and IHC fluorescence.

Paraffin embedding has a long history going back more than a century. GFP labeling technology has accelerated the development of the life sciences, including neuroscience, cancer studies, drug screening, and biosensing applications. Therefore, we believe that the method presented herein provides a valuable bridge between two important methods that were previously incompatible. This may enable new types of studies and future breakthroughs. For example, paraffin embedding may provide a convenient way to combine pathological diagnosis and fluorescently labeled tumors. This method can also bring about a new level of understanding for both basic and clinical, as well as preclinical studies.

## Data Availability

All datasets generated for this study are included in the manuscript and/or the Supplementary Files. The raw data supporting the conclusions of this manuscript will be made available by the authors, without undue reservation, to any qualified researcher.

## Ethics Statement

The protocol was approved by the Committee on the Ethics of Animal Experiments of the Huazhong University of Science and Technology (Permit Number: 00027340). All surgery was performed under sodium pentobarbital anesthesia, and every effort was made to minimize animal suffering.

## Author Contributions

XL and HG conceived and designed the project. OZ performed the experiments. PZ took charge of the viral labeling. XY and YY performed part of the data acquisition. XL and OZ wrote the manuscript.

## Conflict of Interest Statement

The authors declare that the research was conducted in the absence of any commercial or financial relationships that could be construed as a potential conflict of interest.
