# Supplementary material for: Maintenance of Fluorescence During Paraffin Embedding of Fluorescent Protein-Labeled Specimens
Source: Front Neurosci. 2019 Jul 23;13:752. doi: 10.3389/fnins.2019.00752 (PMC6664058; doi:10.3389/fnins.2019.00752)
Supplement: Supplementary file 1 [file Table_1.DOCX]

Supplementary Material

## Supplementary Figures

##
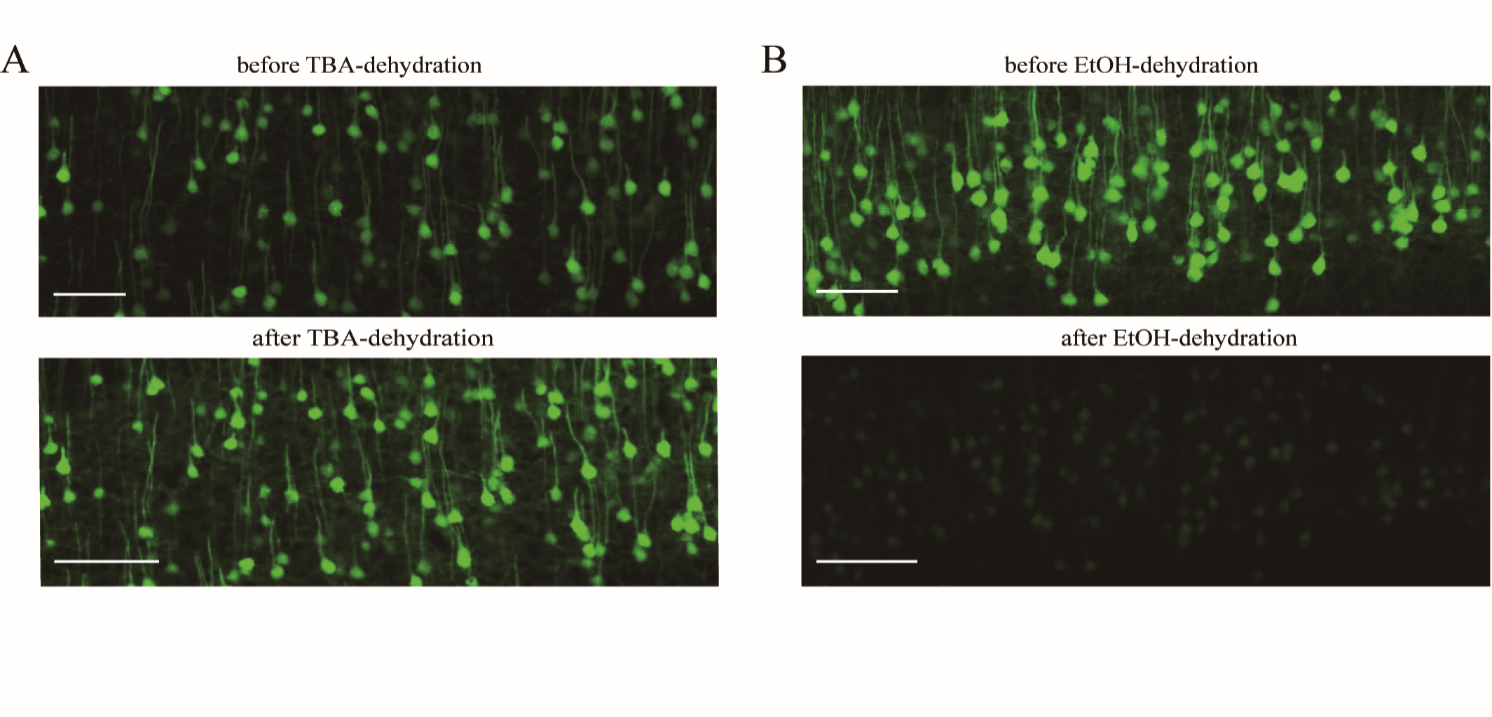


Supplementary Figure 1. 100μm-thick fresh brain sections were collected and dehydrated as the following. Dehydration steps: (50%, 75%, 95%，100% ,100% and a third 100% V/V, each incubated for 1h at 30°C). All the pictures were imaged by LSM 710 with the same parameters. **(A)** Images of fluorescent sections before and after dehydrating by TBA. **(B)** Images of fluorescent sections before and after dehydrating by EtOH. Scale bars, (A, B) 100μm; red box.

**
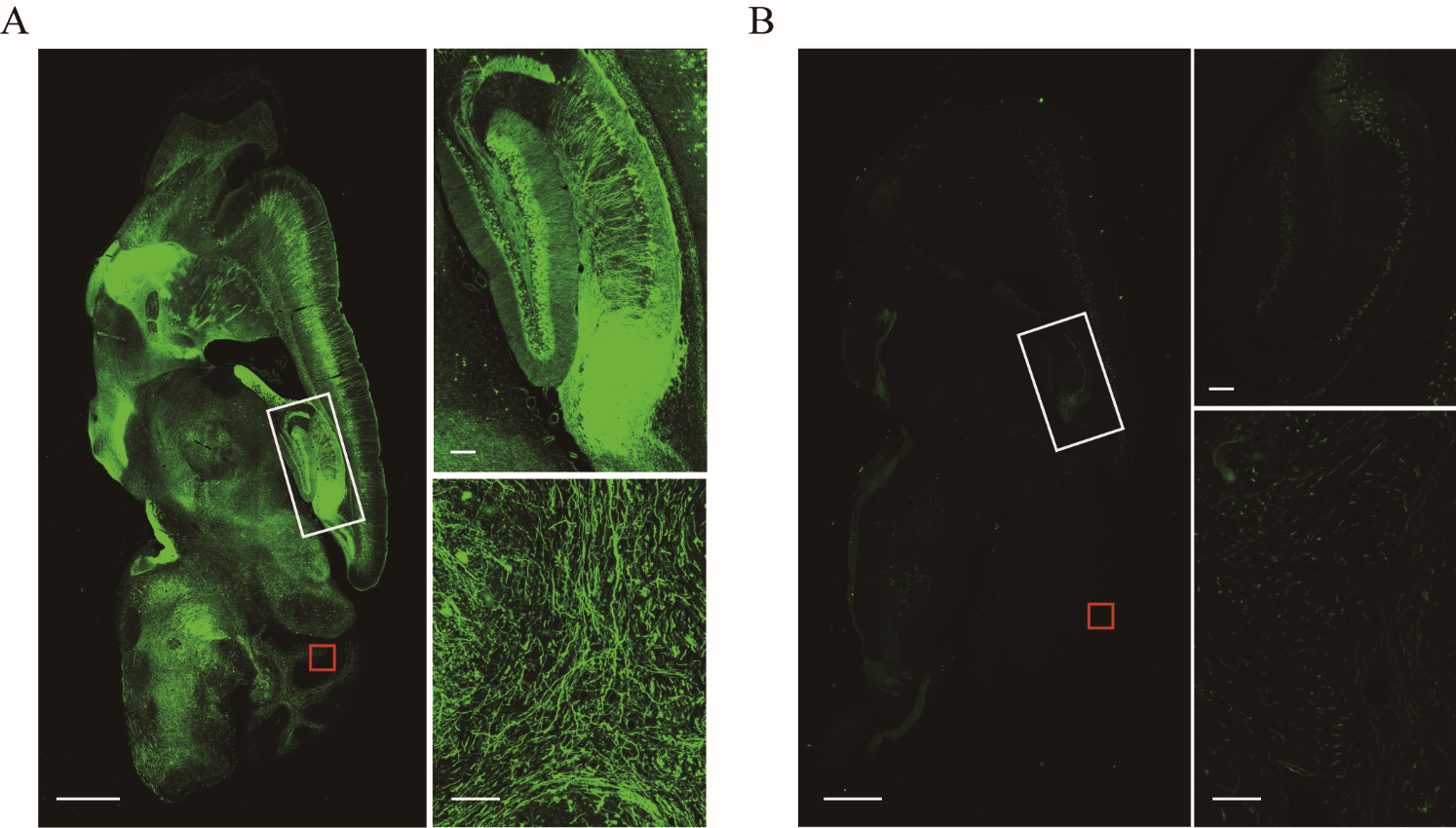
**

Supplementary Figure 2. **(A)** Images of paraffin-embedded fluorescent hemispheres. The left hemisphere was dehydrated with EtOH and embedded with paraffin. **(B)** Images of paraffin-embedded fluorescent hemispheres. The right hemisphere was dehydrated with TBA and then embedded with paraffin. Scale bars, (A, B) 1000μm; white box, 100μm; red box, 50μm.


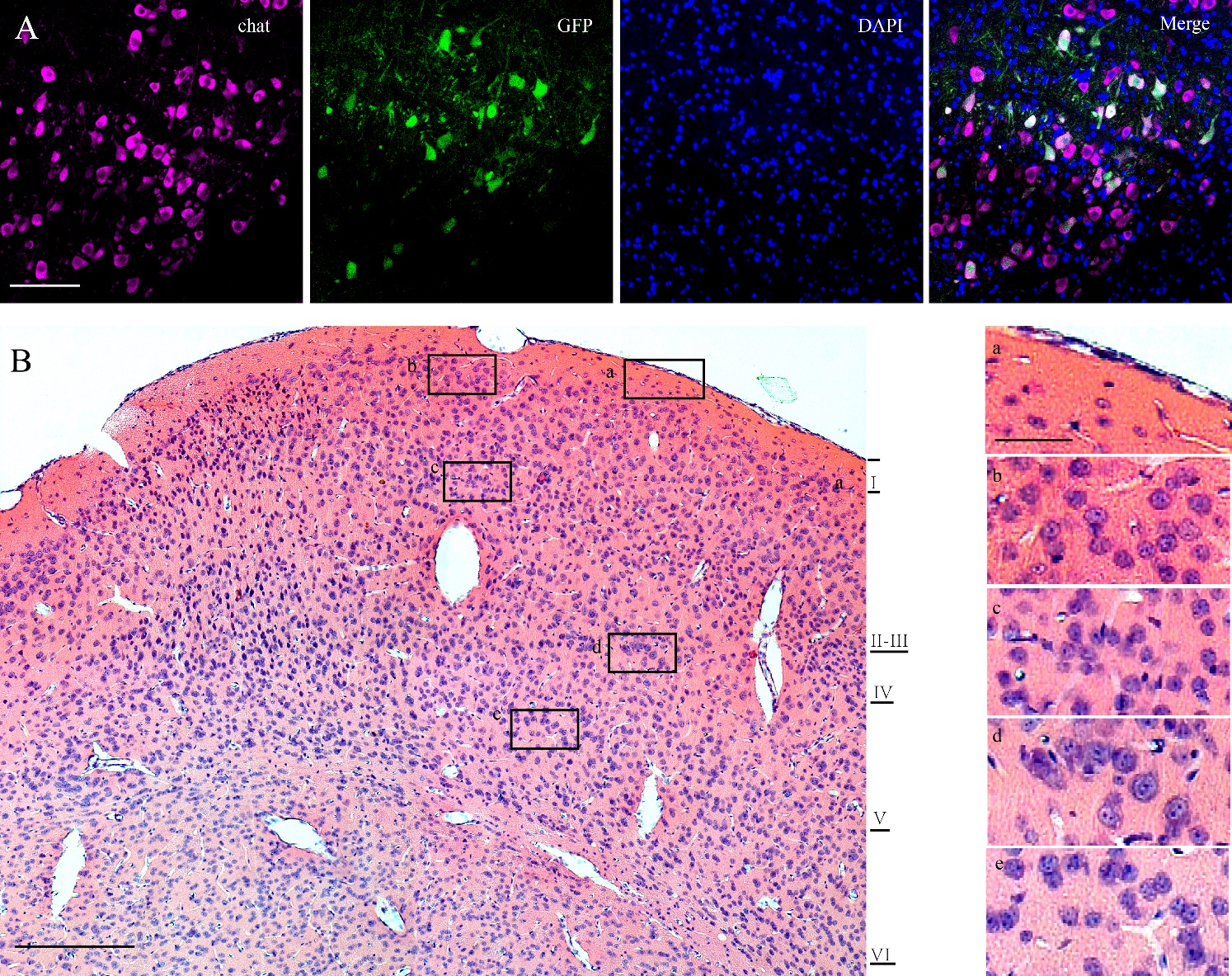


Supplementary Figure 3. **(A)** Images of paraffin section stained by HIC. **(B)** Images of paraffin section stained by H&E. Scale bars, (A) 100μm; (B) 500μm, black box, 50μm.
